# Abscisic Acid (ABA) Regulation of *Arabidopsis* SR Protein Gene Expression

**DOI:** 10.3390/ijms151017541

**Published:** 2014-09-29

**Authors:** Tiago M. D. Cruz, Raquel F. Carvalho, Dale N. Richardson, Paula Duque

**Affiliations:** Instituto Gulbenkian de Ciência, Rua da Quinta Grande 6, Oeiras 2780-156, Portugal; E-Mails: tcruz@igc.gulbenkian.pt (T.M.D.C.); rcarvalho@igc.gulbenkian.pt (R.F.C.); drichardson@igc.gulbenkian.pt (D.N.R.)

**Keywords:** abscisic acid (ABA), ABA-responsive *cis* elements, alternative splicing (AS), *Arabidopsis thaliana*, gene expression, SR proteins

## Abstract

Serine/arginine-rich (SR) proteins are major modulators of alternative splicing, a key generator of proteomic diversity and flexible means of regulating gene expression likely to be crucial in plant environmental responses. Indeed, mounting evidence implicates splicing factors in signal transduction of the abscisic acid (ABA) phytohormone, which plays pivotal roles in the response to various abiotic stresses. Using real-time RT-qPCR, we analyzed total steady-state transcript levels of the 18 SR and two SR-like genes from *Arabidopsis thaliana* in seedlings treated with ABA and in genetic backgrounds with altered expression of the ABA-biosynthesis *ABA2* and the ABA-signaling *ABI1* and *ABI4* genes. We also searched for ABA-responsive *cis* elements in the upstream regions of the 20 genes. We found that members of the plant-specific SC35-Like (SCL) *Arabidopsis* SR protein subfamily are distinctively responsive to exogenous ABA, while the expression of seven SR and SR-related genes is affected by alterations in key components of the ABA pathway. Finally, despite pervasiveness of established ABA-responsive promoter elements in *Arabidopsis* SR and SR-like genes, their expression is likely governed by additional, yet unidentified *cis*-acting elements. Overall, this study pinpoints *SR34*, *SR34b*, *SCL30a*, *SCL28*, *SCL33*, *RS40*, *SR45* and *SR45a* as promising candidates for involvement in ABA-mediated stress responses.

## 1. Introduction

Precursor messenger RNA (pre-mRNA) splicing is an essential step in gene expression mediated by the spliceosome, a large protein complex in the cell nucleus that interacts with specific intronic sequences in the pre-mRNA called splice sites for the proper removal of introns and correct joining of exons. Alternative splicing (AS) occurs when splice sites are differentially recognized, allowing for the production of multiple transcripts from a single gene that can potentially result in different protein isoforms. In addition to largely expanding the coding capacity of genomes, AS represents an important means of regulating gene expression, for instance by introducing premature termination codons (PTCs) that then target these transcripts for degradation by a process known as nonsense-mediated mRNA decay (NMD) [1].

Serine/arginine-rich (SR) proteins constitute a highly conserved family of RNA-binding proteins (RBPs) with key roles in constitutive, and particularly AS, by affecting splice site selection in a concentration- and phosphorylation-dependent manner [2]. Their distinctive protein domain organization consists of one or two *N*-terminal RNA recognition motifs (RRMs) and an arginine/serine-rich domain (RS) located at the *C*-terminus [3]. The RRM binds the pre-mRNA conferring target specificity [4] whereas the RS domain, which can be highly phosphorylated at multiple serine residues [5], is involved in protein-protein interactions allowing the recruitment of core components of the splicing machinery [4,6]. SR proteins act by binding auxiliary *cis*-acting regulatory elements, such as exonic or intronic splicing enhancers or silencers (ESE/ESS; ISE/ISS), thereby influencing splice site choice and bridging spliceosomal components at the 5' and 3' splice sites [7]. Additional roles have been uncovered in animal systems for these splicing factors, including in mRNA nuclear export, NMD and translation, or in genome stability, transcriptional elongation and microRNA processing [7,8,9,10].

Despite considerable progress in recent years, the functions of plant SR proteins remain less understood than those of their metazoan counterparts. The *Arabidopsis thaliana* genome codes for 18 SR proteins that can be grouped into six subfamilies. The SR, RSZ and SC subfamilies include direct orthologs of the mammalian SR splicing factors SRSF1, SRSF7 and SRSF2, respectively, while the SCL, RS2Z and RS subfamilies are plant-specific. Additionally, the *Arabidopsis* genome encodes two SR-like proteins, SR45 and SR45a, which despite sharing properties with other SRs display an atypical domain organization (two RS domains flanking an RRM) that does not meet the accepted SR protein nomenclature criteria [11]. A few *Arabidopsis* SR and SR-like proteins have been shown to function as *bona fide* splicing factors [12,13] and to play *in vivo* roles in plant development and/or responses to environmental cues [13,14,15,16,17]. Importantly, several have also been found to target AS of multiple pre-mRNAs, including those of other members of the SR gene family [13,14,15,18,19].

AS is pervasive in higher eukaryotes and has long been known to determine key biological processes in animal systems [20,21]. In humans, about 95% of the multi-exon genes undergo AS [22,23], of which misregulation is associated with numerous severe diseases [24,25]. In higher plants, the first transcriptome-wide analyses indicate that half or more of the intron-containing genes are alternatively-spliced [26,27], with the current estimate in *Arabidopsis* plants grown under normal conditions being above 60% [28]. However, the biological relevance of AS in plants is just beginning to unfold, namely in developmental processes, as well as in the response to pathogens and, particularly, abiotic stress [29,30,31]. In fact, stress-associated genes are particularly prone to AS [32,33,34], which is markedly affected by abiotic stresses [32,35,36,37,38,39], and the expression/splicing pattern of several SR or SR-like proteins is stress-regulated [38,40,41,42], pointing to a crucial role for AS in plant responses to environmental stress.

Abscisic acid (ABA) is a major plant hormone playing a key role as an abiotic stress response modulator. Indeed, ABA is crucial in the coordination of several signal transduction pathways involved in the response to environmental stresses such as drought, high salinity, cold and heat, all of which are known to increase endogenous ABA levels [43,44]. Currently, the core ABA signaling network is comprised of 13 pyrabactin resistance 1-like (PYL) receptors, nine A-type protein phosphatase 2Cs (PP2Cs), three sucrose nonfermenting-1-related protein kinase 2s (SNRK2s) and five basic leucine zipper transcriptional activators known as ABA-responsive element binding factors (ABFs) [45,46]. However, recent systems biology approaches have uncovered an ABA signaling network of over 500 interactions among 138 different proteins [47]. Transcriptional activation of downstream genes responsive to ABA is governed by phosphorylation of the ABFs, which bind to *cis* elements in target gene promoters known as ABREs (ABA-responsive elements) [45] to activate gene expression. While the core ABFs play an important role in ABRE-dependent gene expression, many genes induced by ABA lack ABRE elements and the transcription factors that activate these genes have yet to be clearly delineated [47].

Interestingly, emerging evidence is linking plant RBPs, and splicing factors in particular, to ABA-mediated stress responses. The conserved splicing factor Suppressor of *abi3-5* (SUA) was found to influence seed ABA sensitivity in *Arabidopsis* by regulating AS of the *ABI3* signaling gene [48]. Similarly, a recent study showed that AtSF1, the *Arabidopsis* homolog of the mammalian SF1 splicing factor, is active in AS and regulates sensitivity to ABA [49]. ABA-related phenotypes have also been described for mutants in plant heterogeneous nuclear ribonucleoproteins (hnRNPs) [50,51,52], another class of RBPs active in AS. Regarding the SR protein family, Palusa *et al*. [40] showed that exogenous ABA application slightly alters the AS pattern of three *Arabidopsis* SR genes, and Xiong and co-workers [17] have identified ABA and salt stress hypersensitive phenotypes upon deletion of two members of the plant-specific RS subfamily, *RS40* and *RS41*. A knockout mutant for a member of the SCL subfamily, *SCL30a*, is also hypersensitive to ABA during seed germination (our unpublished results). Finally, the SR-like SR45 protein negatively regulates glucose signaling during early seedling development in *Arabidopsis* by downregulating the ABA pathway [16]. These findings suggest that posttranscriptional networks may act as central coordinators of plant abiotic stress responses by targeting key components of the ABA signal transduction machinery.

To investigate the extent of SR gene expression regulation by the stress phytohormone ABA, we have analyzed total transcript levels of the 20 *Arabidopsis thaliana* SR and SR-like genes by real-time RT-qPCR in plants treated exogenously with ABA and in different ABA-related genetic backgrounds. Furthermore, to gain insight into which SR and SR-like genes may respond to the core ABFs, we also examined upstream sequences for each of the SR and SR-like genes for occurrences of putative ABRE *cis* elements.

## 2. Results and Discussion

### 2.1. Effect of Exogenous Abscisic Acid (ABA) on the Expression of Arabidopsis Serine/Arginine-Rich (SR) and SR-Like Genes

**Figure 1:**
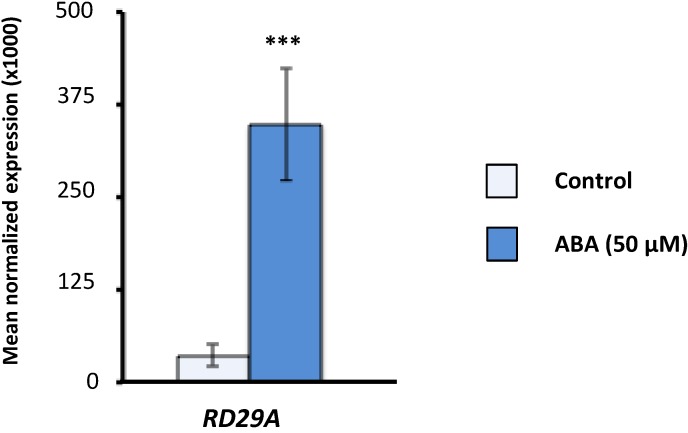
Effect of the abscisic acid (ABA) treatment on *RD29A* gene expression. Real-time RT-qPCR analysis of total transcript levels of the ABA-responsive gene *RD29A* in control and ABA-treated (50 µM for 5 h) *Arabidopsis* seedlings. Results are from two independent experiments and values represent means ± SE (*n* = 4). Asterisks indicate a significant difference (*** *p* < 0.001; Student’s *t*-test) from control plants.

To study the effect of the stress phytohormone ABA on total steady-state transcript levels of SR and SR-like genes, we treated 15 day-old *Arabidopsis* seedlings from the Columbia (Col-0) ecotype with 50 μM ABA for 5 h and performed real-time RT-qPCR. To ensure that the treatment was effective, RT-qPCR analyses were first performed with primers specific to the ABA-responsive gene *RD29A* [53]. Indeed, as seen in Figure 1, a six-fold increase in *RD29A* expression levels was observed, indicating that the ABA treatment worked.

**Figure 2:**
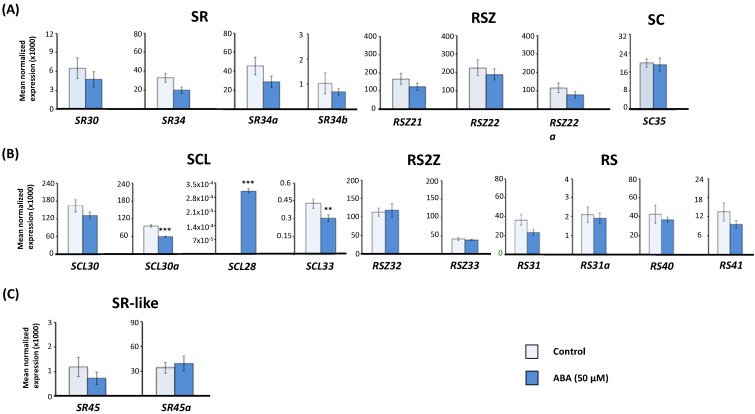
Effect of ABA treatment on the expression of *Arabidopsis* serine/arginine-rich (SR) and SR-like genes. Real-time RT-qPCR analysis of total transcript levels of SR gene members of the mammalian ortholog (**A**) and plant-specific (**B**) subfamilies or of SR-like genes (**C**) in control and ABA-treated (50 µM for 5 h) wild-type (Col-0) *Arabidopsis* seedlings. Results are from two independent experiments and values represent means ± SE (*n* = 4). Asterisks indicate significant differences (** *p* < 0.01, *** *p* < 0.001; Student’s *t*-test) from the corresponding wild type.

As shown in Figure 2A,B, among the 18 *Arabidopsis* SR genes, only three members of the plant-specific SCL subfamily (*SCL30a*, *SCL28* and *SCL33*) had their expression patterns significantly altered (*p* < 0.05). While *SCL28* expression was induced by ABA, *SCL33* and *SCL30a*—Two gene paralogs located on duplicated regions of the genome [54,55]—Were repressed (Figure 2B). The expression profile of these three SR genes suggests they could very likely be involved in ABA responses. In agreement with our results, the *SCL33* gene has been previously described as being downregulated by ABA but, contrary to what we report, *SCL28* and *SCL30a* were found to be unaffected by ABA treatment [40]. Previous studies using whole genome tiling arrays have shown that ABA treatments for different periods of time activate different genes [56,57], and this could explain why *SCL30a* and *SCL28* behaved differently in our study, as not only the ABA concentration but also the time points used were different from those reported by Palusa *et al.* [40]. On the other hand, the induction seen in *SCL28* transcript levels may also be the result of a more accurate quantification through real-time RT-qPCR, as the expression levels for this gene are very low and can hardly be detected by semi-quantitative RT-PCR. Given that none of the members from the other SR gene subfamilies exhibited misregulation upon ABA treatment, the plant-specific SCL subfamily may be distinctly responsive to this stress phytohormone.

Regarding the SR-like genes, neither *SR45* nor *SR45a* gene expression was affected by the exogenous application of ABA (Figure 2C). Indeed, the expression of *SR45* has been reported to show significant changes under sucrose and temperature stress [58], but not in response to ABA or other phytohormones [40,58]. Expression of *SR45a* is markedly induced by high light stress [59], while DNA microarray studies [60,61,62,63] and RNA-seq analysis of abiotic stress samples [64] have shown that *SR45a* mRNA abundance is also altered upon heat and water deprivation stresses. Nevertheless, *SR45a* expression in response to ABA was not investigated until this study.

### 2.2. Expression of Arabidopsis SR and SR-Like Genes in the aba2-1 Mutant Background

**Figure 3:**
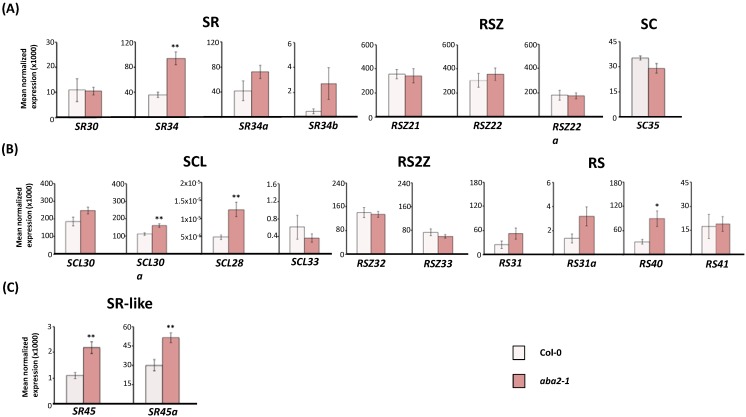
Expression of *Arabidopsis* SR and SR-like genes in the ABA-deficient *aba2-1* mutant background. Real-time RT-qPCR analysis of total transcript levels of SR gene members of the mammalian ortholog (**A**) and plant-specific (**B**) subfamilies or of SR-like genes (**C**) in wild-type (Col-0) and *aba2-1* mutant seedlings. Results are from two independent experiments and values represent means ± SE (*n* = 4). Asterisks indicate significant differences (* *p* < 0.05, ** *p* < 0.01; Student’s *t*-test) from the corresponding wild type.

After studying the effect of exogenously applied ABA on the expression levels of SR and SR-like genes, we next asked whether a reduced endogenous plant ABA content would affect these genes. To address this question, we compared total SR and SR-like transcript levels in wild-type (Col-0) seedlings and those from the ABA-deficient mutant *aba2-1* [65] (Figure 3). The *ABA2* gene encodes a short-chain alcohol dehydrogenase/reductase (SDR) protein that catalyzes the conversion of xanthoxin to abscisic aldehyde in the ABA biosynthesis pathway, and its loss of function leads to substantially reduced ABA levels [65,66,67,68].

From the mammalian ortholog *Arabidopsis* SR subfamilies, only expression of the *SR34* gene was altered in *aba2-1*, displaying higher steady state transcript levels in this mutant background (Figure 3A). Although still not functionally characterized, the *SR34* gene was previously reported to be repressed by exogenous ABA [40]. That *SR34* expression was upregulated in the *aba2-1* mutant lends further credence to the notion that this SR gene is modulated by the phytohormone.

Three SR plant-specific genes were affected in the *aba2-1* mutant background: *SCL30a* and *SCL28*, members of the SCL subfamily, and *RS40* from the RS subfamily (Figure 3B). All three genes exhibited higher transcript levels in the mutant when compared to the wild type. The upregulation of the *SCL30a* gene in *aba2-1* seedlings is concordant with its repression by ABA treatment (see Figure 2B) and with previously produced data in our lab supporting SCL30a as a negative regulator of ABA-dependent salt and drought stresses during seed germination (unpublished results). According to the *Arabidopsis* electronic Fluorescent Pictograph (eFP) browser [69], *SCL28* expression is low at almost all phases of the *Arabidopsis* life cycle. Intriguingly, this SR gene was induced both by exogenous ABA (see Figure 2B) and in the *aba2-1* mutant background. This may indicate that *SCL28* expression is under tight ABA control, with slight changes in endogenous basal hormone levels being sufficient to disrupt this regulation. The third plant-specific SR gene affected in the *aba2-1* mutant, *RS40*, was very recently implicated in the ABA pathway. In fact, knockout mutants for *RS40* or its duplicated gene pair *RS41* were found to display ABA hypersensitivity during seed germination and early seedling development [17]. The same study reports that expression of both these SR genes is slightly ABA induced, but their transcript levels were not significantly changed by the phytohormone under our experimental conditions (see Figure 2B).

Finally, expression of the two SR-related genes, *SR45* and *SR45a*, was noticeably induced in the *aba2-1* mutant background (Figure 3C). A loss of function mutant for SR45, *sr45-1*, is hypersensitive to glucose and, similarly to the *rs40* and *rs41* mutants, also displays an oversensitive response to the exogenous application of ABA [16]. Moreover, the *sr45-1* mutation causes enhanced glucose-specific ABA accumulation, as well as ABA biosynthesis and signaling gene expression [16]. The observation that *SR45* is upregulated in plants impaired in ABA synthesis suggests that ABA represses the gene’s expression *in vivo*. This could be important for amplification of ABA signal transduction under stress conditions, where enhanced production of ABA would reduce the levels of its negative regulator SR45, thereby releasing repression of ABA-mediated inhibition of early seedling development. Future detailed characterization of the *Arabidopsis* *SR45a* gene, which was also markedly upregulated in the *aba2-1* background, should reveal whether it fulfills similar ABA-related functions.

### 2.3. Expression of Arabidopsis SR and SR-Like Genes in the abi1-1, abi4-101 and ABI4OX Backgrounds

**Figure 4:**
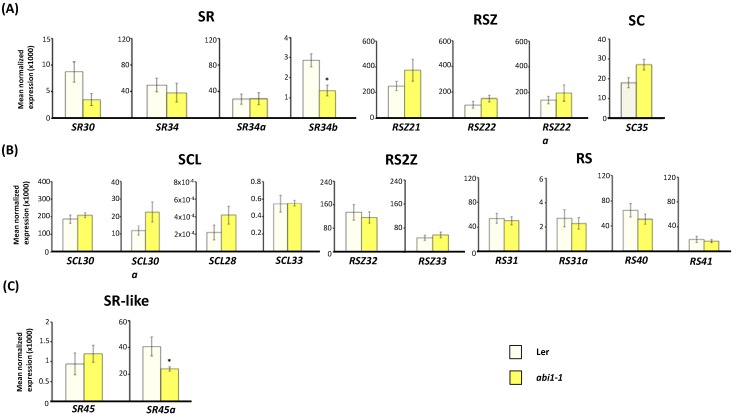
Expression of *Arabidopsis* SR and SR-like genes in the ABA-signaling *abi1-1* mutant background. Real-time RT-qPCR analysis of total transcript levels of SR gene members of the mammalian ortholog (**A**) and plant-specific (**B**) subfamilies or of SR-like genes (**C**) in wild-type (L*er*) and *abi1-1* mutant seedlings. Results are from two independent experiments and values represent means ± SE (*n* = 4). Asterisks indicate significant differences (* *p* < 0.05; Student’s *t*-test) from the corresponding wild type.

**Figure 5:**
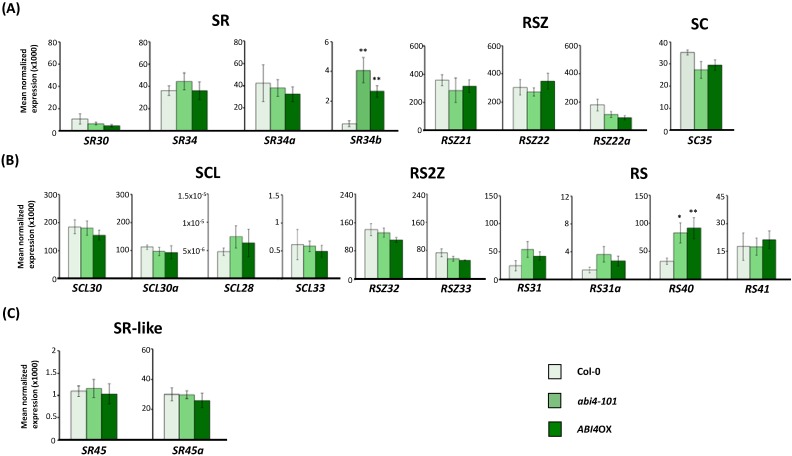
Expression of *Arabidopsis* SR and SR-like genes in the ABA-signaling *abi4-101* mutant and *ABI4*OX transgenic backgrounds. Real-time RT-qPCR analysis of total transcript levels of SR gene members of the mammalian ortholog (**A**) and plant-specific (**B**) subfamilies or of SR-like genes (**C**) in wild-type (Col-0), *abi4-101* mutant and *ABI4*OX transgenic seedlings. Results are from two independent experiments and values represent means ± SE (*n* = 4). Asterisks indicate significant differences (* *p* < 0.05, ** *p* < 0.01; Student’s *t*-test) from the corresponding wild type.

In addition to exogenous ABA application and reduced ABA biosynthesis, we also investigated if alterations in the levels of ABA signaling components could affect SR and SR-like gene expression. To evaluate this hypothesis, we checked the total transcript levels of *Arabidopsis* SR and SR-like genes in the ABA-insensitive *abi1-1* [70] and *abi4-101* [71] mutants, as well as in an *ABI4*-overexpressing (*ABI4*OX) transgenic line [72] (Figure 4 and Figure 5). The *ABI1* gene encodes protein phosphatase 2C (PP2C), a membrane protein that plays a key role in ABA sensing and signaling [73,74]. ABI4 is a versatile ethylene responsive factor/APetala2 (ERF/AP2) transcription factor essential for ABA-dependent transcriptional changes that acts both as an activator and a repressor of gene expression [75]. Both genes mediate ABA signal transduction but while *ABI4* function has been primarily described in seed tissues and during early seedling development, *ABI1* is also known to play crucial roles at the vegetative phase namely in the regulation of stomatal closure.

As depicted in Figure 4A,B, of all the *Arabidopsis* SR genes only *SR34b* exhibited altered expression in the *abi1-1* mutant (*p* < 0.05). In fact, although several RBP-encoding genes were found to be misregulated in a genomewide expression profiling of *Arabidopsis* wild-type (L*er* ecotype) versus *abi1-1* mutant seedlings, no changes in SR or SR-like genes were noticed [76]. Our observed repression of *SR34b* in the *abi1-1* background suggests that expression of this gene is under *ABI1*-signaling control, pointing to a role in ABA-mediated responses. Indeed, and although here total *SR34b* expression was unaffected by ABA treatment (see Figure 2), Palusa *et al.* [40] reported that AS of the *SR34b* pre-mRNA is altered by exogenous ABA and environmental stresses. Interestingly, the *Arabidopsis* splicing factor SUA was found to control ABA-mediated seed responses by specifically regulating AS of an another ABI signaling gene, *ABI3* [48]. Whether *SR34b* plays any role in splicing of the *ABI1* pre-mRNA remains to be elucidated—What our results do indicate is that ABI1 is involved in promoting expression of the SR34b splicing factor.

As for the two *Arabidopsis* SR-like genes, while *SR45* expression remained unchanged, *SR45a* was downregulated nearly two fold in the *abi1-1* mutant background (Figure 4C). This again strongly suggests that *SR45a* expression is targeted by ABI1-mediated signaling, holding much promise for the future functional characterization of this SR-related gene in ABA responses.

Finally, we analyzed the expression patterns of *Arabidopsis* SR and SR-like genes in the *abi4-101* and *ABI4*OX backgrounds (Figure 5). We found no changes in the total transcript levels of any of the SR or SR-like genes in seedlings of either *ABI4* genotype (*p* < 0.05), except for the *SR34b* gene, belonging to the SR subfamily of orthologs of the mammalian splicing factor SRSF1, and for the plant-specific *RS40* gene (Figure 5A,B). Surprisingly, both loss and gain of *ABI4* function led to induction of *SR34b* and *RS40* expression.

The observed induction of *SR34b* and *RS40* in the transgenic *ABI4*OX background suggests a role for the ABI4 transcription factor in the activation of expression of these SR genes. However, we also observed an increase of *SR34b* and *RS40* expression levels in the *abi4-101* mutant. Given that severe mutations in the *ABI4* gene result in relatively weak phenotypes, it has been proposed that defects in *ABI4* function may be masked by genetic redundancy [77], in which case proteins with overlapping functions could compensate for a reduction in ABI4 levels and explain the sustained induction of the *SR34b* and *RS40* genes in the *abi4-101* mutant. Alternatively, upregulation of *RS40* and *SR34b* in both the *abi4-101* mutant and *ABI4* overexpressing lines may indicate that these SR genes are under tight ABI4 control and any fluctuations in the levels of this versatile transcription factor are sufficient to trigger their induction. In either case, our results point to the SR34b and RS40 splicing factors as promising candidates for involvement in ABI4-related functions, such as regulation of seed dormancy/germination [72], sugar signaling [78], lateral root development [79] or defense responses [80].

### 2.4. ABA-Responsive cis Elements in Upstream Sequences of Arabidopsis SR and SR-Like Genes

**Figure 6:**
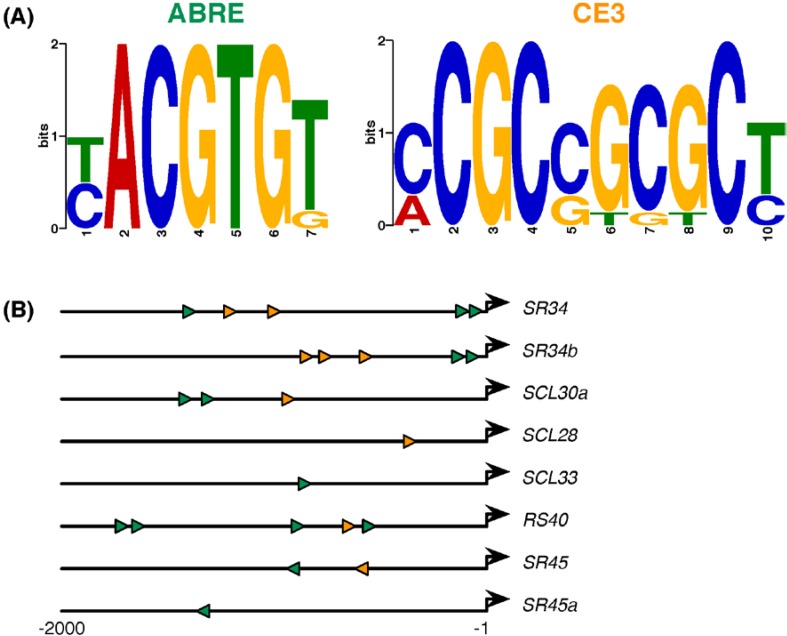
ABA-responsive *cis*-element consensus sequences and their locations/orientations in the eight SR/SR-like genes responsive to ABA and/or alterations in the ABA pathway. (**A**) The ABRE (7 bp) and CE3 (10 bp) motif logos as generated by MEME [86] based on previously reported motifs [84]; and (**B**) Schematic representation of the relative position and orientation of ABRE (green) and CE3 (orange) elements found in the 2 kb upstream sequences of the SR or SR-like genes found to respond to any of the ABA scenarios analyzed (see Figure 2, Figure 3, Figure 4 and Figure 5). The bold black arrows denote the transcription start sites. Exact coordinates of the elements are presented in Table 1.

To better understand SR and SR-like gene expression regulation by exogenous ABA or alterations in ABA pathway components, we examined 2 kb of upstream sequence for each of the *Arabidopsis* 20 SR and SR-like genes for occurrences of previously reported ABA-responsive *cis* elements. We focused our attention on two well-characterized elements shown to confer responsiveness to ABA: the ACGT-containing ABRE and the functionally equivalent non-ACGT containing element known as Coupling Element 3 (CE3) [81,82,83,84,85] (Figure 6A).

As described in the Experimental Section, we scanned the 2-kb upstream sequences of each of the 20 SR or SR-like genes for significant occurrences of ABRE and CE3 motifs. Table 1 consolidates the results of our *cis*-element analysis and indicates the number of each motif found, their locations and orientations in the upstream regions, and whether the gene in question responded to any of the ABA scenarios described above (see Figure 2, Figure 3, Figure 4 and Figure 5).

**Table 1:** ABA-responsive *cis* elements in 2 kb upstream sequences and corresponding expression changes under various ABA scenarios of *Arabidopsis* SR and SR-like genes. For each gene, the corresponding number of ABA-responsive elements (ABRE) and coupling elements (CE3) (*p* < 0.0001), their locations within 2 kb of upstream sequence and whether the gene responds to ABA or genetic alterations of ABA pathway components are presented. The (+) and (−) signs next to the gene names denote the chromosomal gene orientation. Motif locations are presented as start coordinates upstream of the translation start site; ABRE locations are shown in green, whereas CE3 locations are presented below in orange. Superscripts to the right of the motif locations indicate if the motif was found on the forward (+) or reverse (−) strand of the upstream sequence. Upward- and downward-pointing arrows indicate significant induction or repression of gene expression, respectively, in response to various ABA scenarios (see Figure 2, Figure 3, Figure 4 and Figure 5).

Gene	# ABRE	# CE3	Motif Locations (Start)	ABA (50 μM)	*aba2-1*	*abi1-1*	*abi4-101* & ABI4:OX
*RSZ21*(−) (AT1G23860)	3	1	−593^+^, −676^+^, −1460^−^	×	×	×	×
			−681^−^				
*RSZ22* (+) (AT4G31580)	2	0	−166^+^, −522^+^	×	×	×	×
*RSZ22a* (+) (AT2G24590)	1	0	−1844^−^	×	×	×	×
*SC35* (−) (AT5G64200)	1	3	−857^+^	×	×	×	×
			−745^+^, −1009^−^, −1876^−^				
*SR30* (−) (AT1G09140)	1	1	−1968^+^	×	×	×	×
			−405^−^				
*SR34* (+) (AT1G02840)	3	2	−9^+^, −18^+^, −1394^+^	×	↑	×	×
			−1056^+^, −1244^+^				
*SR34a* (+) (AT3G49430)	1	0	−1276^−^	×	×	×	×
*SR34b* (+) (AT4G02430)	2	3	−49^+^, −58^+^	×	×	↓	↑
			−608^+^, −780^+^, −806^+^				
*SCL30* (+) (AT3G55460)	0	2	−1182^+^, −1481^+^	×	×	×	×
*SCL30a* (−) (AT3G13570)	2	1	−1364^+^, −1452^+^	↓	↑	×	×
			−979^+^				
*SCL28* (−) (AT5G18810)	0	1	−353^+^	↑	↑	×	×
*SCL33* (+) (AT1G55310)	1	0	−834^+^	↓	×	×	×
*RSZ32* (−) (AT3G53500)	2	3	−664^+^, −1761^+^	×	×	×	×
			−222^−^, −402^−^, −804^+^				
*RSZ33* (−) (AT2G37340)	0	1	−1852^+^	×	×	×	×
*RS31* (−) (AT3G61860)	3	3	−188^+^, −743^−^, −1286^−^	×	×	×	×
			−1037^−^, −1343^+^, −1393^+^				
*RS31a* (−) (AT2G46610)	2	4	−277^+^, −570^−^	×	×	×	×
			−280^−^, −614^+^, −1453^+^, −1464^−^				
*RS40* (+) (AT4G25500)	4	1	−581^+^, −882^+^, −1686^+^, −1737^+^	×	↑	×	↑
			−692^+^				
*RS41* (+) (AT5G52040)	0	3	−876^+^, −1695^+^, −1892^−^	×	×	×	×
*SR45* (−) (AT1G16610)	1	1	−939^−^	×	↑	×	×
			−628^−^				
*SR45a* (−) (AT1G07350)	1	0	−1317^−^	×	↑	↓	×

Of the 20 SR and SR-like genes analyzed, each gene’s upstream region had at least one of the *cis* elements present. A total of 30 ABRE and 30 CE3 elements were detected, with 25/30 ABREs and 26/30 CE3s falling within the first 1.5 kb of the upstream sequence. While it was previously reported that CE3 elements were sparse in the *Arabidopsis* genome [84], the relatively high occurrence of CE3 elements found here suggests that either the initial assessment of CE3 was underestimated or that the results presented here may be an overestimate. In either scenario, the disparity is likely due to a divergence in methodology: Here we used the program Find Individual Motif Occurrences (FIMO) [87], which calculates log-likelihood ratio scores for each motif position in a given sequence and converts these scores to *p*-values, whereas previously [84] EMBOSS profit [88] was used, which does not calculate *p*-values and relies instead upon arbitrary user-defined score cutoff values. The majority of the ABRE (22/30) and CE3 (19/30) elements were found on the forward strand of the upstream sequences.

Previous studies have suggested that a single occurrence of an ABRE is insufficient for inducing gene expression in response to ABA and that at least another ABRE or coupling element is required for ABA responsiveness [85,89]. Interestingly, the sole gene induced by exogenous ABA treatment was *SCL28* (Table 1; see also Figure 2), which harbors only a single coupling element in its upstream region (Figure 6B; Table 1). In line with this, six late embryogenesis abundant (*LEA*) genes in *Prunus mume* were found to be induced by ABA, but all six corresponding promoters lacked ABREs, highlighting the likelihood that there are other, yet unidentified, ABA-responsive elements important in regulating gene expression in the presence of ABA [90]. The upregulation of *SR34*, *SCL30a*, *SCL28*, *RS40*, *SR45* and *SR45a* in the *aba2-1* mutant (Table 1; see also Figure 3) is also suggestive of other *cis* elements and/or transcription factors mediating their ABA responsiveness. The reasoning here is that ABFs typically bind to ABREs or coupling elements to induce gene expression in response to enhanced ABA levels, and the observed upregulation of the six genes in a background with a reduced ABA content suggests their repression by the phytohormone. Similarly, the downregulation of *SCL33* and *SCL30a* upon exposure to exogenous ABA (Table 1; see also Figure 2) could be mediated by as yet unknown transcriptional repressors. Up to 11 different families of transcription factors are implicated in the response to ABA-related processes [91], including WRKYs that have been shown to inhibit transcription from the promoter of specific ABA-responsive genes [92,93]. In addition to transcription factors, ABA-mediated gene expression can also be regulated by receptors, secondary messengers, protein kinase/phosphatase cascades and chromatin-remodeling factors [91]. In agreement with this, *SR34b* (two ABRE and three coupling elements) and *SR45a* (one ABRE) were unresponsive to exogenous ABA treatment but were downregulated in the *abi1-1* mutant (Table 1; see also Figure 2 and Figure 4), which is defective in a PP2C membrane protein phosphatase critical in ABA sensing. Lastly, *SR34b* and *RS40* expression was altered in the *ABI4*-related genetic backgrounds (Table 1; see also Figure 5). Each of these SR genes has a total of five ABRE/CE3 elements in their upstream regions that could be responsible for the observed expression changes (Figure 6B and Table 1). The observed upregulation of *SR34b* and *RS40* in both the *abi4-101* mutant and *ABI4* overexpressor is puzzling. It may be that the dual roles of ABI4 as both an activator and repressor of transcription [75] are at play in the ABA-mediated regulation of *SR34b* and *RS40* gene expression. A CCAC motif has been proposed to function as the principal ABI4-binding element in genes downregulated by this transcription factor [94,95]. Indeed, *SR34b* contains CCAC motifs significantly enriched in its upstream region; however, this motif was not found in the upstream region of *RS40* (data not shown). It is nonetheless plausible that another ABI4 binding site could confer negative regulation of *RS40* expression. Taken together, these findings highlight the importance of conducting linker-scanning mutagenesis to conclusively discern the contribution of particular *cis* elements to ABA-regulated SR and SR-like gene expression. The eight genes (Figure 6B) whose expression was affected by ABA and/or alterations in ABA components provide a suitable starting point for such analyses.

## 3. Experimental Section

### 3.1. Plant Materials and Growth Conditions

The *Arabidopsis thaliana* ecotypes Colombia (Col-0) or Landsberg *erecta* (L*er*) were used as the wild type in this study. The *aba2-1* and *abi4-101* (Col-0 background) or *abi1-1* (L*er* background) mutants were obtained from the Nottingham *Arabidopsis* Stock Centre (Nottingham, UK). The *ABI4*OX line (Col-0 background) [72] was kindly provided by Qi Xie (Chinese Academy of Sciences, Beijing, China). Seeds from the different genotypes were surface-sterilized for 10 min in 50% (*v*/*v*) bleach and 0.07% (*v*/*v*) Triton X-100, stratified for three days at 4 °C in the dark (to break dormancy) and plated on MS medium (1× Murashige and Skoog (MS) salts (Duchefa Biochemie, Haarlem, The Netherlands), 2.5 mM 4-morpholineethanesulfonic acid (MES) (pH 5.7), 0.5 mM myo-inositol and 0.8% (*w*/*v*) agar), before transfer to a growth chamber under long-day (16 h light; 80 µmol·m^−2^·s^−1^ white light) conditions at 60% relative humidity and 22 °C (light period)/18 °C (dark period). For all analyses, plant material was collected 15 days after stratification.

### 3.2. ABA Treatment

To analyze the effect of ABA treatment on SR and SR-related gene expression, 15-day old seedlings were transferred to new MS medium plates supplemented with 50 μM ABA (mixed isomers, A1049; Sigma-Aldrich, St. Louis, MO, USA), with control plants being transferred to MS plates without ABA. After 5 h the plant material was harvested and frozen in liquid nitrogen for subsequent RNA extraction. The effectiveness of the ABA treatment was confirmed by RT-qPCR using *RD29A* as a marker gene (see Figure 1).

### 3.3. RNA Extraction and Reverse Transcription

Total RNA was extracted from whole seedlings using the innuPREP Plant RNA kit (Analytik Jena BioSolutions, Jena, Germany) following the protocol provided. All RNA samples were digested with DNAse I (Promega, Madison, WI, USA) and phenol-chloroform purified. First strand cDNA was then synthesized using 1 µg of RNA, oligo-(d)T primer and M-MLV Reverse Transcriptase (Promega) according to the manufacturer’s instructions.

### 3.4. Real-Time RT-qPCR Analyses

Real-time RT-qPCR was performed using a CFX 384 Touch Real-Time PCR Detection System (Bio-Rad, Hercules, CA, USA) and the Luminaris Color HiGreen High Rox qPCR Master Mix (Thermo Scientific, Waltham, MA, USA) on 2.5 µL of cDNA (diluted 1:10) per 10 µL reaction volume, containing 300 nM of each gene-specific primer (Table 2). Primers were designed to span a common region shared by all alternative transcripts annotated in TAIR and/or reported in Palusa *et al.* [40] and, thus, detect total expression of each SR or SR-related gene. The reaction was initiated by a Uracil-DNA Glycosylase step (50 °C for 2 min), cycles were 95 °C for 10 min (1×), 95 °C for 15 s/60 °C for 30 s/72 °C for 30 s (40×), followed by a melting curve step to confirm the specificity of the amplified products. For each condition tested, two independent biological and technical repetitions were performed, as described by Remy *et al.* [96]. Data were processed using Q-Gene [97] that took the respective primer efficiency into consideration. Statistical analysis was performed using R software (http://www.r-project.org/), Student’s *t*-test was used, and a two-sided *p* value lower than 0.05 was accepted to indicate statistical significance.

### 3.5. ABA cis-Regulatory Element Analysis

For each of the 20 SR/SR-like genes analyzed, 2 kb of upstream sequence was acquired from the upstream sequences dataset at TAIR (http://www.arabidopsis.org). This dataset is comprised of upstream sequences that begin either from the 5'-UTR if the gene has an annotated 5'-UTR or from the translation start site if no such annotation exists. However, in our dataset, all upstream sequences began from the transcription start site of the representative gene model for each SR or SR-like gene in TAIR. The upstream sequences were scanned using the FIMO [87] tool of the Multiple Expectation Maximization for Motif Elicitation MEME Suite [86] web server. Motif position weight matrices used for scanning were constructed using MEME [98] on the high scoring ABA-responsive element (ABRE) and coupling element (CE3) motifs previously reported [84]. Motif occurrences were considered significant if they had a *p*-value <1 × 10^−4^. If a motif occurred as a palindrome, it was counted only once. If a high scoring motif had multiple instances and these were due to a shift of a few base pairs (*i.e.*, a subsequence of one another), only the highest scoring motif occurrence was counted.

**Table 2:** Sequence of the primers used in the RT-qPCR analyses.

	Gene	Primers	Sequence (5'–3')
**ABA Marker**	*RD29A*	RD29AqF1	AACGACGACAAAGGAAGTGG
	(At5g52310)	RD29AqR1	CATCCTTTAATCCTCCCAACC
**SR Subfamilies**			
**SCL**	*SCL30*	SCL30qF2	GAAGCAGATACCGATCAAGGTC
	(At3g55460)	SCL30qR2	CTCATTGTCTCCATTTCTGTCC
	*SCL30a*	SCL30aqF2	AGATTCCAGGACAGAAGACG
	(At3g13570)	SCL30aqR2	TGATGTCTTTTAGCGGGAGG
	*SCL28*	SCL28qF3	AGGCGAGAGTCAAGGCATAGTA
	(At5g18810)	SCL28qR3	GGCAAAGGAGAACGTGAAATAG
	*SCL33*	SCL33qF2	ATCTATCTCGCCCAGGGAAG
	(At1g55310)	SCL33qR2	GGCTCTTACCTCTGACTGGAGTT
**RS2Z**	*RSZ32*	RSZ32qF1	CCAAAGGTAGGGACCAAAGC
	(At3g53500)	RSZ32qR1	GCAGAGTTCCTGCCATTACC
	*RSZ33*	RSZ33qF1	GAGGAGAGATCACGCAGTCC
	(At2G37340)	RSZ33qR1	GCTCCCGTCTATGATCTTTGG
**RS**	*RS31*	RS31qF2	CCCGAGAAGGTCTCTTAGTCC
	(At3g61860)	RS31qR2	CTGTCGTATTCTGGGCTTCG
	*RS31a*	RS31aqF2	ATGCTGGCAGTCGAAGAAGG
	(At2g46610)	RS31aqR2	AGGAGCAGGACCCTTGTACC
	*RS40*	RS40qF3	CCGTTCAAGAAGGAGAGTCC
	(At4g25500)	RS40qR1	TTTCAACTTGGCCATTCTCG
	*RS41*	RS41qF2	GGTCAAGGTCGAAGTCAAGC
	(At5g52040)	RS41qR2	GCTCCATCGTATCCTCTTCC
**RSZ**	*RSZ21*	RSZ21qF2	AGGCGTAGAAGCCCTAGTCC
	(At1g23860)	RSZ21qR2	GCGAGGTGGAGTAACACTGC
	*RSZ22*	RSZ22qF1	CCGCAGCTACAGTAGATCACC
	(At4g31580)	RSZ22qR1	TCTGCGTCTTTCTTTCAGTCC
	*RSZ22a*	RSZ22aqF1	CCTCCAAGACGTCGTAGTCC
	(At2g24590)	RSZ22aqR1	CTGCGCACATCTTTCAGACC
**SC**	*SC35*	SC35qF3	CACAGTCGTTCCTTGAGTGC
	(At5g64200)	SC35qR3	TGGAGACCTCTCATTGCTACG
**SR**	*SR30*	SR30qF2	GACCGTAAAGGCATGTCTGG
	(At1g09140)	SR30qR2	TTCAGTGGCATCAAGTTTCC
	*SR34*	SR34qF1	AAGGCAAAGTCTTCACGTAGG
	(At1g02840)	SR34qR1	AGACGGTGACCTTGACTTCG
	*SR34a*	SR34aqF2	GCAGAAGCAGAAGCAGAAGC
	(At3g49430)	SR34aqR2	ATCGATCTGGAAAGGGATCG
	*SR34b*	SR34bqF4	TTGAGGATGCTCGTGATGC
	(At4g02430)	SR34bqR4	GTTCCACCCGTAAATGATGC
**SR-like**	*SR45*	SR45qF2	GCGATCACCTGATTCTCCC
	(At1g16610)	SR45qR2	AGATCTATATCGTCTTGGAGG
	*SR45a*	SR45aqF1	TGACCGATCATGCTCACCC
	(At1g07350)	SR45aqR1	CTGTGCCTTCTGTAGTAACG

## 4. Conclusions

Given that many pre-mRNA splicing factors are beginning to be implicated in ABA-mediated stress responses, we screened the 20 *Arabidopsis thaliana* genes encoding SR or SR-related proteins for total expression changes induced by exogenous ABA treatment or in different genetic backgrounds with alterations in key ABA pathway components.

ABA treatment of *Arabidopsis* seedlings notably only affected the expression of SR genes belonging to the plant-specific SCL subfamily. In fact, three of the four SCL members had their transcript levels significantly altered, indicating that they may be direct targets of ABA-responsive transcriptional activators (*SCL28*) or repressors (*SCL30a* and *SCL33*). It should be noted, however, that gene regulation upon exogenous ABA application does not necessarily imply ABA regulation *in vivo*. Conversely, that expression of the vast majority of SR and SR-related genes was not directly affected by ABA treatment does not exclude the possibility that they function in ABA-related responses. For example, the SR45 splicing factor has been shown to negatively regulate sugar signaling through downregulation of the ABA pathway [16]. Nevertheless, *SCL30a*, which we have found is involved in ABA-mediated salt and osmotic stress responses during seed germination (unpublished results), was both repressed by exogenous ABA and induced in the ABA-deficient *aba2-1* mutant background, strongly suggesting its transcriptional repression by the phytohormone.

In addition to *SCL30a*, the expression of another five genes was altered in *aba2-1*, the ABA scenario in which changes in more SR and SR-like genes were observed. As this mutant background is known to display reduced endogenous ABA levels [65,66,67,68], this setting may provide a more accurate assessment of direct *in vivo* regulation of gene expression by the stress hormone. Apart from two members of the SCL subfamily, *SCL30a* and *SCL28*, another plant-specific SR gene, *RS40*, and the mammalian ortholog *SR34*, as well as the two *Arabidopsis* SR-related genes, *SR45* and *SR45a*, exhibited altered expression in *aba2-1* mutant seedlings. Interestingly, all six genes were upregulated, suggesting ABA-mediated repression of their steady-state transcript levels, although the intriguing observation that *SCL28* is also markedly induced by exogenous ABA application requires further investigation.

Fewer genes were found to have their expression changed by alterations in the levels of the ABA signaling components ABI1 and ABI4. While the *SR34b* mammalian ortholog was repressed and induced in the ABA-insensitive *abi1-1* and *abi4-101* mutant backgrounds, respectively, the plant-specific *RS40* and *SR45a* genes were only changed (up- and downregulated, respectively) in the *abi4-101* or the *abi1-1* mutant. Surprisingly, both genes found to be induced in the *abi4-101* background, *SR34b* and *RS40*, were also upregulated in transgenic *Arabidopsis* plants overexpressing ABI4, possibly owing to the ability of this transcription factor to act both as an activator and repressor of gene expression [75].

Though all *Arabidopsis* SR and SR-like genes were found to harbor at least one ABA-responsive *cis* element in their upstream regions and some contain up to six elements, our results show that the mere presence alone of such elements does not translate directly into ABA-responsive gene regulation. It is likely that many as yet undiscovered *cis* elements play a role in governing ABA-mediated SR gene expression. Overall, this study indicates that six SR genes and the two SR-like genes from *Arabidopsis thaliana* are likely involved in ABA-related responses. While *SR34b*, *RS40* and *SR45a* are most likely downstream targets of the ABI1 PP2C phosphatase and the ABI4 transcription factor, the ABA responsiveness of the *SR34*, *SCL30a*, *SCL28*, *SCL33* and *SR45* genes appears to be independent of these ABA signaling components.

It should be noted that the present study focused on total SR/SR-like gene transcript levels and hence potential effects of ABA treatment or alterations in the ABA pathway on the AS profiles were not analyzed. The *Arabidopsis* SR gene family is known to undergo extensive AS—Having been estimated to generate about 95 mRNAs [40]—Which is likely to account for significant gene expression regulation. Furthermore, many of these alternative transcripts contain PTCs, pointing to an important posttranscriptional role of NMD in fine-tuning the abundance of functional SR gene transcripts present in the cell [35,99]. Although a previous semi-quantitative RT-PCR analysis did not detect major ABA-induced changes in the splicing pattern of *Arabidopsis* SR genes [40], further studies leveraging the discriminatory power of RNA-seq will facilitate not only an isoform-specific analysis of ABA regulation of SR and SR-related genes, but also allow for a full transcriptomic/differential AS snapshot of the plant’s response to ABA. Moreover, it will be possible to assess the downstream effect of ABA within the context of posttranscriptional regulation of SR genes, e.g., ABA-induced NMD of SR isoforms. Last but not least, posttranslational modifications may also play a key role in ABA regulation of plant SR proteins. Indeed, aside from the well-established phosphorylation of RS domains to regulate protein-protein interactions and subcellular localization of SR proteins [100], several other modifications such as lysine acetylation, arginine methylation, ubiquitination or SUMO conjugation have been reported in animal systems to regulate SR protein degradation, stability and localization [101]. The regulation of SR gene expression is thus a decidedly complex affair involving multiple levels of control, and the mechanisms underlying ABA-regulated expression of plant SR/SR-like proteins, as well as their involvement in the ABA pathway, await further study.
